# Effect of hypoxia-inducible factor-prolyl hydroxylase inhibitors on anemia in patients with CKD: a meta-analysis of randomized controlled trials including 2804 patients

**DOI:** 10.1080/0886022X.2020.1811121

**Published:** 2020-09-01

**Authors:** Bin Wang, Qing Yin, Yu-Chen Han, Min Wu, Zuo-Lin Li, Yan Tu, Le-ting Zhou, Qing Wei, Hong Liu, Ri-Ning Tang, Jing-Yuan Cao, Lin-Li Lv, Bi-Cheng Liu

**Affiliations:** a Institute of Nephrology, Zhong Da Hospital, Southeast University School of Medicine, Nanjing, China; b Department of Nephrology, Wuxi People’s Hospital, Wuxi, China

**Keywords:** HIF-PHIs, renal anemia, chronic kidney disease, meta-analysis

## Abstract

Hypoxia-inducible factor-prolyl hydroxylase inhibitors (HIF-PHIs) are orally active first-in-class new generation drugs for renal anemia. This extensive meta-analysis of randomized controlled trials (RCTs) was designed to provide clear information on the efficacy and safety of HIF-PHIs on anemia in chronic kidney disease (CKD) patients. Searches included PubMed, Web of Science, Ovid MEDLINE, and Cochrane Library database up to October 2019. RCTs of patients with CKD comparing HIF-PHIs with erythropoiesis-stimulating agents (ESAs) or placebo in the treatment of anemia. The primary outcome was hemoglobin change from baseline (Hb CFB); the secondary outcomes included iron-related parameters and the occurrence of each adverse event. 26 trials in 17 articles were included, with a total of 2804 dialysis or patients with CKD. HIF-PHIs treatment produced a significant beneficial effect on Hb CFB compared with the placebo group (MD, 0.69; 95% CI, 0.36 to 1.02). However, this favored effect of HIF-PHIs treatment was not observed in subgroup analysis among trials compared with ESAs (MD, 0.06; 95% CI, −0.20 to 0.31). The significant reduction in hepcidin by HIF-PHIs was observed in all subgroups when compared with the placebo group, whereas this effect was observed only in NDD-CKD patients when compared with ESAs. HIF-PHIs increased the risk of nausea (RR, 2.20; 95% CI, 1.06 to 4.53) and diarrhea (RR, 1.75; 95% CI, 1.06 to 2.92). We conclude that orally given HIF-PHIs are at least as efficacious as ESAs treatment to correct anemia short term in patients with CKD. In addition, HIF-PHIs improved iron metabolism and utilization in patients with CKD.

## Introduction

Anemia is a common complication in patients with chronic kidney disease (CKD) and is associated with poor clinical outcome [1–3]. Correcting anemia can reduce mortality, hospitalization, and improve the quality of life in patients with CKD [4–8]. Current guidelines and recommendations for anemia management in patient with CKD is recombinant human erythropoietin (rhEPO) and its analogs (called erythropoiesis-stimulating agents, ESAs), supplemented with intravenous iron administration [9]. Although rhEPO could markedly correct anemia, supraphysiologic EPO concentrations achieved during rhEPO treatment may contribute to the adverse cardiovascular effects [10–12]. In addition, the elevation of blood pressure caused discontinuation, hyporesponsiveness due to inflammation or iron depletion were potential causes of unsatisfied anemia control in patients with patients with CKD undergoing rhEPO treatment [13–15]. In addition, ESAs require adequate iron supplementation, which may place patients at increased risk of allergic reactions, infections, and cardiovascular events [12,16]. Thus, novel therapeutic strategies are necessary for the improved anemia in patients with CKD.

Currently, the introduction of HIF-PHIs into clinical practice might have a revolutionary influence on anemia treatment due to their unique pharmacological effects [17]. So far, seven different PHIs including Roxadustat (35 trials), Vadadustat (30 trials), Daprodustat (37 trials), Molidustat (15 trials), Enarodustat (6 trials), Desidustat (1 trial), and DS-1093a (2 trials) are being investigated in more than 100 clinical trials [18]. On 17 December 2018, Roxadustat was licensed to treat anemia in China after successfully finishing its Phase III studies [19]. Numerous studies have consistently shown a better effect of different HIF-PHIs than placeboand at least as efficacious as classic rhEPO treatment to correct anemia in patients with CKD who already undergo dialysis or not [20–28]. However, inconsistent results still existed in the degree to which hemoglobin was changed between different or even in the same HIF-PHIs treatment [20–28]. Of note, the relatively small sample sizes are the common limitations mentioned in all clinical trials (confined to phase II or III) about HIF-PHIs on anemia treatment. Hence, we first conducted a meta-analysis of randomized controlled trials (RCT) of all these seven HIF-PHIs to determine their effects on the correction of anemia, regulation of iron metabolism, and the incidence of adverse events.

## Methods

We performed and reported our meta-analysis in line with the PRISMA (Preferred Reporting Items for Systematic Reviews and Meta-analyses) guidelines [29].

### Literature search and study selection

We searched English language publications up to 07 October 2019 on the following databases and international and national clinical trial registries: Ovid Medline, PubMed, Web of Science and the Cochrane Library database (no date restriction), with relevant text words and medical subject headings that included all spellings of “Prolyl hydroxylase inhibitor”, “PHD inhibitor”, “hypoxia-inducible factor stabilizer”, “HIF stabilizer”, “Roxadustat”, “FG-4592”, “Vadadustat”, “AKB-6548”, “Daprodustat”, “GSK127883”, “Molidustat”, “BAY 85-3934”, “Enarodustat”, “JTZ-951”, “DS-1093a”, “Desidustat” and “anemia OR anemia”.

Both exclusion and inclusion criteria were prespecified. RCT studies must meet several criteria. Firstly, the population is patients with CKD or dialysis patients whose age is > 18 years old. Secondly, intervention: treatment with HIF-PHIs (Roxadustat, Vadadustat, Daprodustat, Molidustat, Enarodustat, Desidustat, and DS-1093a) regardless of dose and duration. Thirdly, the primary outcome was Hb CFB, and the secondary outcomes included the mean change in the hepcidin, ferritin, transferrin, total iron-binding capacity (TIBC), TSAT and serum iron and the occurrence of each adverse event (hypertension, hyperkalemia, cardiovascular events, vascular access thrombosis, headache, vomiting, nasopharyngitis, nausea, and diarrhea).

Retrospective or prospective observational cohort studies were excluded. If they were review articles, animal or cell studies, conference abstracts, editorials were also removed. In addition, phase I and non-randomized phase II studies were excluded. When the same clinical trial appears in multiple articles, or when cases are mixed between publications, the most recent or most complete reporting study, or both, were included. Studies, such as pharmacokinetics, are also excluded. Resolve differences through discussion and consensus. All the included trials represented unique studies.

### Data extraction

Two of us (Q.Y. and M.W) extracted data independently and in duplicate by using a predesigned data collection form, based on the Cochrane handbook for systematic reviews of intervention. Discrepancies were resolved by discussion and consensus, with the senior author (B.C.L.) serving as the final arbiter if consensus could not be reached. The following clinical characteristics for each study were recorded: trial phase, study design, line of treatment, study population, number of patients and mean age in intervention and control groups, follow-up duration, and primary end point (see Table 1). For each prespecified outcome (hemoglobin, hepcidin, ferritin, transferrin, total iron-binding capacity, and serum iron), mean value and standard deviation were extracted. For adverse events, the occurrence counts in each group were extracted. We planned to manage missing data by contacting *via* email the corresponding authors. Where this method was unsuccessful and when the required quantitative data (mean value and standard) were not provided in the literature, g3 data software (www.frantz.fi/software/g3data.php) was used to extract exact numbers from published figures.

**Table 1.** Characteristics of the included trials.

Reference	Trial phase	Design	Trial drugs	Control	Study population	No. of enrolled subjects		Follow-up (weeks)	Mean age (years)		Primary end point
						Intervention	Control		Intervention	Control	
Akizawa et al. [30]	2	Double-blind, placebo-controlled	Daprodustat	Placebo	HD	97	19	8	62.2	63.48	Hb change from baseline (CFB) at week 4
Holdstock et al. [31]	2b	Randomized, controlled, open-label, parallel-group, multicenter	Daprodustat	rhEPO and its analogs	CKD stages 3-5	252	44	28	66.5	65.4	Hb concentration at week 24
Meadowcroft et al. [32]	2b	Randomized, controlled, open-label, parallel-group, multicenter	Daprodustat	Placebo	HD	216	177	28	59.6	59.7	Hb change from baseline at week 4
Holdstock et al. [23]	2a	Randomized, controlled parallel-group, multicenter	Daprodustat	Placebo	CKD stages 3–5	73	19	6	68.3	69.2	Modeled change in Hb from baseline over 4 weeks of treatment
				rhEPO	HD	83	21	6	55.7	64.2	
Martin et al. [27]	2a	Multicenter, randomized, double-blind	Vadadustat	Placebo	CKD stages 3-4	191	19	6	65.7	64.9	Mean absolute change in Hb from baseline to the end of treatment
Pergola et al. [20]	2b	Double-blind, randomized, placebo-controlled	Vadadustat	Placebo	nondialysis dependent CKD	138	72	20	66.6	65.9	The percentage of patients achieved or maintained a mean Hb level of 11.0 g/dl or an increase in Hb of 1.2 g/dl over the predose average (average of the 2 Hb values obtained before dosing at screening and baseline) during the last 2 weeks of treatment
Akizawa et al. [25]	2	Randomized, double-blind	Roxadustat	Placebo	non-dialysis-dependent CKD	80	27	24	64.4	61.9	The rate of rise in Hb from baseline to the final assessment in the fixed-dose period (EOF)
Besarab et al. [34]	2a	Single-blind, randomized placebo-controlled dose-ranging and pharmacodynamics	Roxadustat	Placebo	non-dialysis-dependent CKD	88	28	12	65.8	68.6	Maximum Hb change from baseline at any time from baseline to 8 weeks of treatment
Chen et al. [35]	2	Double-blinded	Roxadustat	Placebo	CKD stages 3-5, not on dialysis, with baseline Hb < 10g/dl	61	30	8	49.7	51.4	The percentage of subjects with successful dose conversion defined as a Hb level maintained at no <0.5g/dL below mean baseline value during the last 2 weeks of the 6-week dosing period
		Open-label		Epoetin alfa	HD with Hb 9-12g/dl	74	22	6	50.8	53.8	
Chen et al. [22]	3	Randomized, open-label	Roxadustat	Epoetin alfa	HD	305	101	27	47.6	51	Mean change in Hb level from baseline to the average level during weeks 23 through 27
Chen et al. [22]	3	Randomized, double-blind	Roxadustat	Placebo	CKD	154	52	8	54.7	53.2	Mean change from baseline in the Hb level, averaged over weeks 7 through 9
Provenzano et al. [36]	2	Randomized, open-label	Roxadustat	Epoetin alfa	HD	54	13	8	55.8	59.5	The proportion of participants whose Hb levels did not decrease by > 0.5 g/dL from baseline (defined as the mean of the last 3 Hb values obtained prior to the first dose of study treatment)
				Epoetin alfa	HD	90	23	4	56.9	57	The proportion of participants whose mean Hb level was ≥11 g/dL averaged over the last 4 weeks (weeks 16 through 19)
Akizawa et al. [38]	2b	Randomized, double-blind	Enarodustat	Placebo	CKD ESA-naïve patients	94	23	6	NR	65.3	Hb level increase rate per week for the correction group
				Placebo	CKD ESA-treated patients	107	24	6	NR	66.2	The proportion of subjects maintained a change from baseline Hb level within ± 1.0 g/dL at the evaluation point for the conversion group
Akizawa et al. [25]	2b	Randomized, double-blind	Enarodustat	Placebo	HD	121	22	6	NR	60.7	The percentage of subjects with a change in the Hb level within ± 1.0 g/dL from baseline to the evaluation-point
Parmar et al. [24]	2	Multicentric, randomized, double-blind	Desidustat	Placebo	CKD stages 1-4	117	30	6	48.48	46.9	Hb increase in a dose-related manner across all the doses after 6 weeks of treatment
Macdougall et al. [26]	2b	Randomized, double-blind	Molidustat	Placebo	CKD stages 3-5, not on dialysis, ESA-naïve patients, with mean Hb < 10.5g/dl	101	19	12	69	67	The change in Hb level between baseline and the evaluation phase
		Open-label		Darbepoetin	CKD stages 3-5, not no dialysis, stable darbepoetin treated, with mean Hb 9-12g/dl	92	28	12	68	69	
		Open-label		Epoetin	On dialysis, stable epoetin treated, mean Hb 9-11.5g/dL	157	39	13	59	59	
Akizawa et al. [38]	2b	Controlled, parallel-group, open-label, multicenter	Molidustat	Darbepoetin	Not on dialysis	164	42	52	70	69	The change in blood Hb level from baseline to each post-baseline visit during the main phase of the study
				Epoetin	On dialysis	88	30	52	61	59	

HD: hemodialysis; Hb: hemoglobin; rhEPO: recombinant human erythropoietin; CKD: chronic kidney disease; NR: not reported.

### Risk of bias assessment and quality assessment

The Cochrane Risk of Bias Tool was applied to evaluate the risk of bias [30]. We examined every trial and scored it as high, low, or unclear risk of bias to the following criteria: random sequence generation; allocation concealment; blinding of participants and personnel to the study protocol; blinding of outcome assessment incomplete outcome data; and selective reporting (Supplementary Table 1).

Standard domains were used to assess the methodological quality of included trials: allocation concealment (adequate if sequentially labeled, sealed and opaque envelopes or central or pharmacy randomization was used; inadequate when pseudo-randomization was used; unclear in all other cases); blinding of investigators, participants, and outcome assessors; use of intention to treat analysis; completeness of follow-up. Any disagreements were resolved by discussion and consensus.

### Statistical analysis

Mean differences (MDs) or standard mean difference (SMD) as the effect size were used to pool results from all studies that reported changes in hemoglobin, hepcidin, ferritin, transferrin, TIBC, serum iron, and TSAT. Risk Ratio (RR) served as the effect size for the pooled analysis of adverse events. A random-effect model was used for pooled analysis to account for heterogeneity across studies. The heterogeneity between studies was assessed by using the Cochran Q test and quantified by I^2^ statistic. Potential heterogeneity was investigated by comparing summary results obtained from subgroups of studies stratified by intervention in the control group, dialysis status, and follow-up duration. Publication bias was assessed with Egger’s test and Begg’s test. All statistical analyses were performed with the Meta for package in R (x64, version 3.3.3, R Foundation for Statistical Computing, Vienna, Austria).

## Results

### Literature search

**Figure 1.**
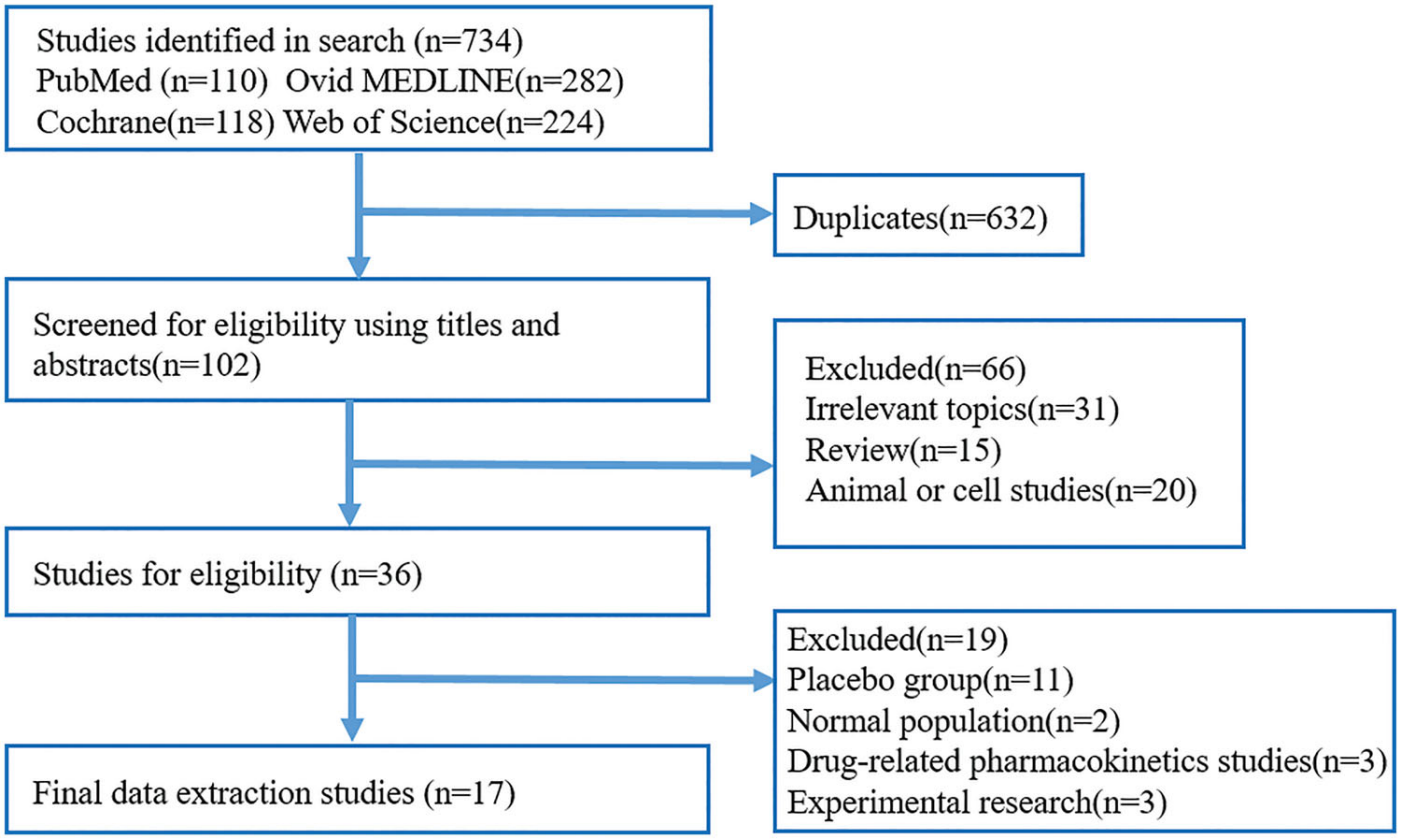
Flowchart diagram of selected randomized controlled trials included in this meta-analysis.

A total of 734 related articles were identified based on the preliminary search strategy. We removed 632 duplicate studies. After screening the titles and abstracts, 66 studies were excluded because they did not meet the inclusion criteria. Then, we carefully reviewed the full text of the remaining 36 eligible papers, 11 of which did not have a placebo group, 2 of which were trials in the normal population, 3 of which were drug-related pharmacokinetics, 3 of which was experimental research. Finally, 17 articles included 26 RCTs were selected for analysis (Figure 1) [20,22–28,31–39]. All eligible research data were obtained from published manuscripts.

### Study characteristics

A total of 2804 subjects from 26 controlled trials in 17 articles were included in this study. Table 1 shows the main characteristics of the eligible trials. Of all the trials studied, 13 trials or subsets included the investigation on the effect of HIF-PHIs on anemic non-dialysis-dependent CKD (NDD-CKD) subjects in 1646 subjects. And 10 trials or subsets contained subsets regarding the effectiveness and safety of HIF-PHIs for the treatment of anemia in 1158 dialysis-dependent CKD (DD-CKD) subjects. Subjects in the intervention arm received daprodustat in 4 studies, vadadustat in 2 studies, roxadustat in 6 studies, enarodustat in 2 studies, desidustat in 1 study, and molidustat in 1 study. All these studies were published between 2015 and 2019. The number of patients recruited in these eligible trials ranged from 67 to 406. And follow-up duration ranged from 4 to 52 weeks. As shown, the mean age in intervened subjects ranged from 47.6 to 70 years and in control subjects ranged from 46.9 to 69 years. Three eligible studies were phase 3 trials, and the other 14 were phase 2 trials. All studies were performed in subjects with anemia. Commonly, the change in Hb during the phase of the study was the primary endpoint for all the eligible trials. The main issue affecting the method quality of the included trials was the lack of blinding because 7 trials were open-labeled (Table 1).

### Quality of the evidence

Concealment of allocation was adequate in only 1 (5.9%) randomized controlled trials, clearly inadequate in 8 (47%) trial, and unclear in the remainder. Participants, investigators, and outcome assessors were blinded in 9 (52.9%) randomized controlled trials, and only 5 (29.5%) randomized controlled trial was analyzed on an intention to treat basis. The dropout rate was less than 10% in 5 (29.4%) trials, between 10%-19% in 8 (47%), 20-39% in 3 (17.6%), and over 40% in only 1 (5.9%) trial.

### Effect of HIF-PHIs on the Hb change from baseline (CFB)

**Figure 2.**
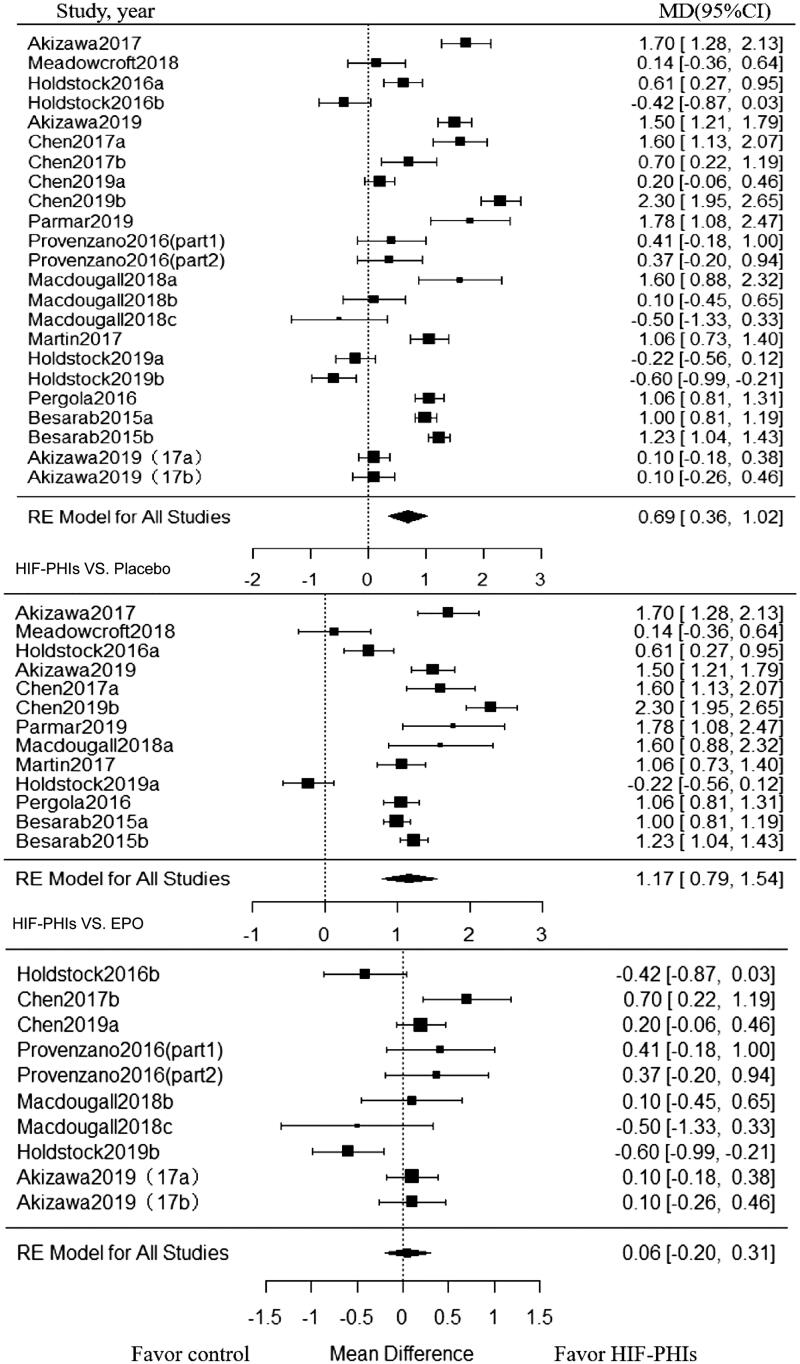
Forest plot for hemoglobin change from baseline. Positive value in mean difference of Hb change represent better anemia correction in PHI group than in the control group. Abbreviations and definitions: MD, mean difference; CI, confidence interval; HIF-PHI, hypoxia inducible factor-Prolyl hydroxylase inhibitor.

Twenty-three trials enrolling 1801 patients, investigated the effect of HIF-PHIs treatment on Hb CFB. HIF-PHIs treatment produced a significant increase in Hb CFB compared with rhEPO or placebo-controlled groups (MD, 0.69; 95% CI, 0.36 to 1.02) (Figure 2). Significant heterogeneity was noted across included trials (I^2^ = 95.02%, *p* < 0.001 for heterogeneity).

Subgroup analysis was further conducted within placebo-controlled trials and rhEPO controlled trials, respectively. In subgroup analysis among placebo-controlled trials stratified by dialysis status and follow-up duration, beneficial effect on Hb CFB associated with HIF-PHIs treatment strengthened in NDD-CKD patients (MD, 1.21; 95% CI, 0.82 to 1.60; I^2^ = 94.35%) and in trials with short follow-up (<20 weeks) (MD, 1.40; 95% CI, 1.06 to 1.75; I^2^ = 89.51%), while such beneficial effect diminished in DD-CKD subjects and trials with long term follow-up (≥ 20 weeks). Notably, among trials compared with rhEPO, the beneficial effect of HIF-PHIs treatment was observed in none of the subgroups. As for different types of HIF-PHIs, the beneficial effect was only limited to Roxadustat in both placebo-controlled trials and rhEPO controlled trials (Table 2).

**Table 2.** Subgroup analysis of hemoglobin by outcome.

Subgroup	No. of trials	MD (95% CI)	I^2^	*p* for heterogeneity test
HIF-PHIs *vs* EPO	10	0.06 (–0.20, 0.31)	70.77%	0.001
Clinical characteristics of participants				
* *HD	7	0.15 (–0.14, 0.44)	63.62%	0.061
* *CKD	3	–0.14 (–0.60, 0.33)	76.27%	0.012
Follow-up weeks				
* *<20 weeks	6	0.14 (–0.25, 0.52)	64.47%	0.011
* *≥20 weeks	4	–0.03 (–0.37, 0.31)	78.51%	0.007
PHI type				
* *Daprodustat	2	–0.52 (–0.82, −0.23)	0%	0.55
* *Roxadustat	4	0.37 (0.11, 0.62)	24.75%	0.343
* *Molidustat	4	0.07 (–0.13, 0.26)	0.01%	0.60
HIF-PHIs *vs* Placebo	13	1.17 (0.79, 1.54)	94.28%	<0.001
* *Clinical characteristics of participants				
* *HD	2	0.93 (–0.60, 2.46)	95.44%	<0.001
* *CKD	11	1.21 (0.82, 1.60)	94.35%	<0.001
Follow-up weeks				
* *<20 weeks	9	1.40 (1.06, 1.75)	89.51%	<0.001
* *≥20 weeks	4	0.63 (–0.15, 1.41)	95.66%	<0.001
* *PHI type				
* *Daprodustat	4	0.56 (–0.26, 1.37)	94.23%	<0.001
* *Roxadustat	5	1.51 (1.07, 1.94)	92.47%	<0.001
* *Molidustat	1	1.60 (0.88, 2.32)	–	1.00
* *Vadadustat	2	1.06 (0.81, 1.31)	0%	1.00
* *Desidustat	1	1.78 (1.08, 2.47)	–	<0.001

HIF-PHI: hypoxia inducible factor-prolyl hydroxylase inhibitor; EPO: erythropoietin; HD: hemodialysis; CKD: chronic kidney disease; MD: mean difference; CI: confidence interval.

### Effect of HIF-PHIs on the change of hepcidin

**Figure 3.**
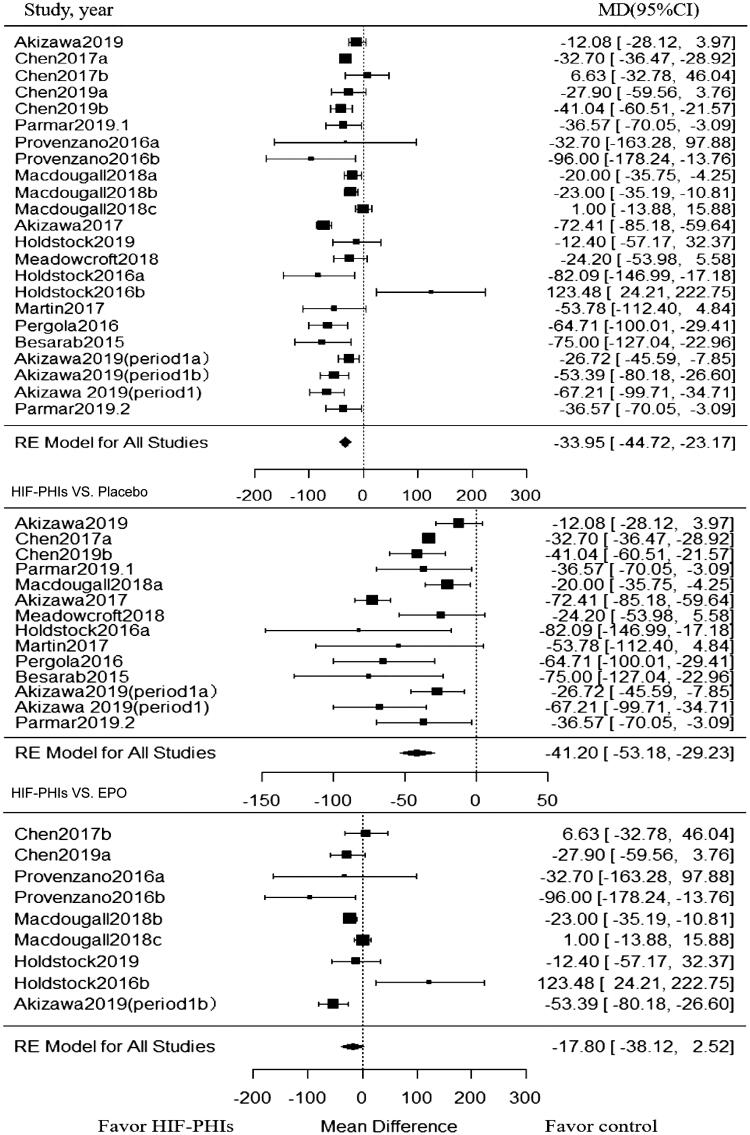
Forest plot for hepcidin change from baseline. Positive value in mean difference of hepcidin change represent a significant lower level of hepcidin in PHI group than in the control group at the end of the treatment of PHI. Abbreviations and definitions: MD, mean difference; CI, confidence interval; HIF-PHI, hypoxia inducible factor-Prolyl hydroxylase inhibitor.

Twenty-three trials enrolling 1866 patients investigated the effect of HIF-PHIs treatment on hepcidin. As showed in Figure 3, when compared with rhEPO or placebo controlled groups, HIF-PHIs treatment induced a significant reduction in hepcidin (MD, −33.95; 95% CI, −44.72 to −23.17). Significant heterogeneity also existed among included trials (I^2^ = 82.2%, *p* < 0.001 for heterogeneity) (Figure 3).

Subgroup analysis was also conducted within placebo-controlled and rhEPO controlled trials, respectively. Among placebo-controlled trials, the significant reduction in hepcidin by HIF-PHIs was observed in all subgroups, and such effect was strengthened in DD-CKD patients (MD, −56.24; 95% CI, −85.69 to −26.78; I^2^ = 76.73%) and in trials with short follow-up (MD, −44.36; 95% CI, −57.39 to −31.32; I^2^ = 78.44%) (Table 3). Among trials compared with rhEPO, the HIF-PHIs induced significant reduction in hepcidin was observed only in NDD-CKD patients (MD, −30.86; 95% CI, −52.99 to −8.73; I^2^ = 56.0%) (Table 3).

**Table 3.** Subgroup analysis of hepcidin by outcome.

Subgroup	No. of trials	MD (95% CI)	*I*^2^	*p* for heterogeneity test
HIF-PHIs *vs* EPO	9	–17.80 (38.12, 2.52)	72.12%	<0.001
Clinical characteristics of participants				
* *HD	6	–6.55 (–47.49, 34.39)	79.64%	0.014
* *CKD	3	–30.86 (–52.99, –8.73)	56.00%	0.103
* *Follow–up weeks				
* *<20 weeks	7	–15.12 (–49.50, 19.26)	88.72%	<0.001
* *≥20 weeks	2	–22.73 (–48.58, 3.12)	0%	0.58
* *PHI type				
* *Daprodustat	2	48.02 (–84.32, 180.36)	83.26%	0.015
* *Roxadustat	4	–26.80 (–65.47, 11.88)	48.64%	0.15
* *Molidustat	2	–11.40 (–34.90, 12.11)	83.28%	0.015
* *Enarodustat	1	–53.39 (–80.18, –26.60)	–	1.00
HIF-PHIs *vs* Placebo	14	–41.20 (–53.18, –29.23)	80.01%	<0.001
* *Clinical characteristics of participants				
* *HD	3	–56.24 (–85.69, –26.78)	76.73%	0.014
* *CKD	11	–32.93 (–42.12, –23.73)	50.14%	0.050
* *Follow-up weeks				
* *<20 weeks	11	–44.36 (–57.39, –31.32)	78.44%	<0.001
* *≥20 weeks	3	–30.58 (–60.29, –0.87)	73.47%	0.029
* *PHI type				
* *Daprodustat	3	–56.82 (–92.65, –21.00)	75.47%	0.012
* *Roxadustat	4	–32.31 (–48.81, –15.81)	74.97%	0.023
* *Molidustat	1	–20.00 (–35.75, –4.25)	–	1.00
* *Enarodustat	2	–44.71 (–84.14, –5.29)	77.57%	0.034
* *Vadadustat	2	–64.71 (–100.01, –29.41)	0%	1.00
* *Desidustat	2	–36.57 (–70.05, –3.09)	–	–

HIF-PHI: hypoxia inducible factor-Prolyl hydroxylase inhibitor; EPO: erythropoietin; HD: hemodialysis; CKD: chronic kidney disease; MD: mean difference; CI: confidence interval.

### Effect of HIF-PHIs on the changes of other iron-related parameters

Twenty-two trials enrolling 1831 patients reported ferritin and 21 trials enrolling 1755 patients reported the TSAT. When compared with rhEPO or placebo, HIF-PHIs significantly reduced ferritin (MD, −0.50; 95% CI, −0.71 to −0.29; I^2^ = 81.48%) and TSAT (MD, −0.38; 95% CI, −0.63 to −0.13; I^2^ = 91.81%) (Supplementary Figure 1 and 5, and Table 4). The same results were obtained in the subgroup analysis of CKD populations (Table 4). 11 trials enrolling 757 patients reported transferrin and 22 trials enrolling 1770 patients reported the TIBC. Both transferrin and TIBC in patients treated with HIF-PHIs were significantly higher than that in rhEPO or placebo groups (For transferrin: MD, 0.91; 95% CI, 0.52 to 1.30; I^2^ = 86.97%; For TIBC: MD, 1.08; 95% CI, 0.83 to 1.34; I^2^ = 86.25%) (Supplementary Figure 2 and 3, and Table 4). The same results were obtained in subgroup analyses of CKD and HD populations (Table 4). Sixteen trials enrolling 1296 patients also compared the serum iron, and no significant difference was observed between the HIF-PHIs group and the control group (MD, 0.09; 95% CI, −0.09 to 0.27; I^2^ = 65.43%) (Supplementary Figure 1–5, and Table 4).

**Table 4.** Subgroup analysis of other iron parameters by outcome.

Subgroup	No. of trials	MD (95% CI)	I 2	*p* for heterogeneity test
Ferritin	22	–0.50 (–0.71, –0.29)	81.48%	<0.001
* *Control drug				
* *Placebo	12	–0.75 (–0.98, –0.51)	67.12%	<0.001
* *EPO	10	–0.20 (–0.46, 0.06)	78.22%	<0.001
* *Clinical characteristics of participants				
* *HD	9	– 0.28 (–0.59, 0.03)	77.43%	<0.001
* *CKD	13	–0.64 (–0.90, –0.38)	80.09%	<0.001
Transferrin	11	0.91 (0.52, 1.30)	86.97%	<0.001
* *Control drug				
* *Placebo	6	1.03 (0.34, 1.73)	91.68%	<0.001
* *EPO	5	0.82 (0.54, 1.09)	43.54%	0.133
* *Clinical characteristics of participants				
* *HD	6	0.93 (0.57, 1.30)	70.32%	<0.001
* *CKD	5	0.89 (0.12, 1.66)	92.56%	<0.001
Total iron binding capacity	22	1.08 (0.83, 1.34)	86.25%	<0.001
Control drug				
* *Placebo	13	1.30 (1.01, 1.58)	78.29%	<0.001
* *EPO	9	0.78 (0.38, 1.17)	87.95%	<0.001
Clinical characteristics of participants				
* *HD	9	1.16 (0.66, 1.65)	90.50%	<0.001
* *CKD	13	1.04 (0.75, 1.33)	82.12%	<0.001
Iron	16	0.09 (–0.09, 0.27)	65.43%	<0.001
* *Control drug				
* *Placebo	8	–0.07 (–0.25, 0.11)	21.36%	0.24
* *EPO	8	0.28 (–0.01, 0.57)	75.04%	<0.001
* *Clinical characteristics of participants				
* *HD	8	0.32 (0.02, 0.62)	72.90%	0.0033
* *CKD	8	–0.13 (–0.27, 0.02)	0%	0.90
Transferrin saturation	21	–0.38 (–0.63, –0.13)	91.81%	<0.001
* *Control drug				
* *Placebo	12	–0.50 (–0.73, –0.28)	65.24%	0.0013
* *EPO	9	–0.19 (–0.69, 0.30)	92.53%	<0.001
* *Clinical characteristics of participants				
* *HD	9	–0.35 (–0.96, 0.26)	94.10%	<0.001
* *CKD	12	–0.41 (–0.53, –0.29)	0%	0.78

EPO: erythropoietin; MD: mean difference; CI: confidence interval.

### Effect of HIF-PHIs on the incidence of adverse events

When compared with the controlled group, HIF-PHIs treated patients experienced significantly more occurrence of nausea (RR, 2.20; 95% CI, 1.06 to 4.52) and diarrhea (RR, 1.75; 95% CI, 1.06 to 2.92) (Table 5). In the placebo-controlled subgroup analysis, HIF-PHIs treated patients experienced significantly more occurrence of nausea (RR, 2.86; 95% CI, 1.13 to 7.24) and hyperkalemia (RR, 2.23; 95% CI, 1.04 to 4.85) (Table 5). No significant differences were found on the incidence of adverse events between HIF-PHIs and EPO group. No significant difference was observed between the HIF-PHIs treated group and the controlled group in the occurrence of hypertension, cardiovascular events, vascular access thrombosis, headache, vomiting, and nasopharyngitis (Table 5).

**Table 5.** Analysis of adverse events (AEs).

Adverse events	No. of trials	Risk ratio (95% CI)	I^2^	*p* for heterogeneity test
Hypertension	12	1 (0.71, 1.40)	0%	0.90
Placebo	6	1.42 (0.70, 2.86)	10.31%	0.61
EPO	6	0.89 (0.59, 1.34)	0%	0.98
Headache	6	1.11 (0.51, 2.41)	0%	0.52
Placebo	5	1.30 (0.58, 2.89)	0%	0.75
EPO	1	0.10 (0.64, 2.44)	–	1.00
Vomiting	6	1.72 (0.69, 4.26)	0%	0.60
Placebo	3	1.12 (0.27, 4.66)	0%	0.83
EPO	3	2.12 (0.52, 8.66)	19.78%	0.26
Nausea	7	2.20 (1.06, 4.52)	0%	0.68
Placebo	4	2.86 (1.13, 7.24)	0%	0.92
EPO	2	0.79 (0.13, 5.00)	27.57%	0.24
Nasopharyngitis	6	1.41 (0.68, 2.94)	24.95%	0.36
Placebo	4	0.86 (0.42, 1.79)	0%	0.76
EPO	1	3.16 (0.41, 24.05)	–	1.00
Diarrhea	12	1.75 (1.06, 2.92)	0%	0.997
Placebo	7	1.90 (0.99, 3.60)	0%	0.97
EPO	4	1.51 (0.54, 4.26)	0%	0.83
Hyperkalemia	8	1.88 (0.80, 4.39)	35%	0.211
Placebo	5	2.23 (1.04, 4.85)	0%	0.85
EPO	2	4.22 (0.57, 31.19)	22.2%	0.26
Cardiovascular events	12	1.22 (0.65, 2.27)	0%	0.94
Placebo	6	1.48 (0.47, 4.71)	0%	0.61
EPO	4	1.14 (0.37, 1.30)	0%	0.81
Vascular access thrombosis	6	0.81 (0.22, 3.00)	0%	0.74
Placebo	3	0.64 (0.09, 4.44)	0%	0.93
EPO	3	0.98 (0.14, 6.82)	16.4%	0.29

CI: confidence interval.

### Publication bias

The Begg rank correlation test and the Egger linear regression test also indicated no evidence of publication bias (Supplementary Table 2).

## Discussion

### Principal findings

We conducted a comprehensive search for trials to compare the efficacy and safety of HIF-PHIs with placebo or EPO in anemic patients with NDD-CKD or DD-CKD. In the present study, we involved 26 trials with 2804 patients and found that HIF-PHIs showed a favored effect than placebo and at least as efficacious as classic rhEPO treatment to correct anemia in patients with CKD in short term. Among different HIF-PHIs, roxadustat even showed a favored effect on Hb CFB than classic rhEPO treatment. In addition, HIF-PHIs caused a reduction in serum hepcidin and ferritin, coupled with increases in transferrin and TIBC, ultimately improving iron utilization.

### Possible explanations

HIF-PHIs exert effects mainly by inhibiting HIF-prolyl hydroxylase enzymes, resulting in the increased expression of HIF target genes. These genes encode proteins, involving in EPO production, iron uptake, mobilization and transport, which then lead to increasing of Hb production and iron mobilization [40,41]. In hypoxic conditions, it also stimulates the expression of the EPO receptor, regulates components of the Hb synthesis pathway, and modulates stem cell maintenance, lineage differentiation, and maturation [42].

The development of renal anemia is predominantly due to a relative deficiency of EPO production by the kidney. However, the supraphysiologic EPO dosing and plasma EPO levels by using of ESAs have been demonstrated to be associated with the increase of cardiovascular morbidity and mortality [10,11,43]. A potentially beneficial feature of HIF-PHI therapy is that Hb targets were achieved with 5- to 17-fold lower plasma EPO levels compared to ESA therapy [24,37]. Therefore, from this point of view, HIF-PHI therapy probably has the potential to improve the cardiovascular outcomes in CKD.

Dysregulation of iron absorption and mobilization also contributed to renal anemia in patients with CKD. In patients with CKD, serum hepcidin is usually high mainly due to the decline of glomerular filtration rate and coexistence of subclinical inflammation, leading to iron uptake and mobilization disorder, and subsequently contributed to the mature hindrance of erythrocytes [44]. Systemic HIF activation suppresses hepcidin production in the liver, enhancing iron uptake and mobilization [45,46]. Hence, the beneficial effect of HIF-PHIs in the treatment of anemia is very unique, just like killing two birds with one stone: increasing expression of EPO within the physiological range and promoting iron utilization.

Although the beneficial effect of HIF-PHIs was diminished in long-term follow-up subgroup analysis, the 95% CI confidence interval was −0.60 to 2.46 in DD-CKD patients and −0.15 to 1.41 in patients with long term follow-up (≧20 weeks). The failure to show an advantage of HIF-PHIs vs. placebo in DD-CKD patients and in patients with long-term follow-up (≧20 weeks) is mainly due to the small number of included studies, resulting in insufficient testing efficiency.

### Clinical implications

Anemia is present in more than 90% of the ESRD patients who undergo dialysis and is a complication that contributes to increased morbidity and mortality [47]. Currently, the standard care for treatment of renal anemia is using of rhEPO and its analogs, supplemented with oral or intravenous (IV) iron administration [48]. However, this strategy poses several clinical challenges and raises multiple patient safety concerns. Firstly, hyporesponsiveness to rhEPO is a major problem affecting 10% patients with CKD, especially in hemodialysis patients [49]; Secondly, use of ESAs in CKD anemia treatment has raised safety concerns as the development or worsening of hypertension, greater risk for death, CV events, and stroke [10,11,13,43], Thirdly, there is a serious concern about the risk and consequences of iron overload in these patients, such as liver toxicity and increased risk of infection [50].

This study indicated that both NDD-CKD and DD-CKD patients could be benefited from different HIF-PHIs in terms of anemia correction, with no obvious and intolerable adverse events. The results suggested that HIF-PHIs as a new class of drug will play an important role in the treatment of renal anemia. While other HIF-PHIs are in phase II and III clinical trials, roxadustat, a first-in-class potent HIF-PHIs, received its first global approval in China on 17 December 2018 for the treatment of anemia in patients with CKD. This makes it possible for HIF-PHIs to be used in renal anemia in patients with CKD. The potential advantages of HIF-PHIs over ESAs in the treatment of renal anemia include: (i) Raising hemoglobin without the risk of increasing in blood pressure (BP); (ii) Reducing the need for iron replacement therapy; (iii) Effective for those resistant to ESAs due to microinflammation; (iv) Orally given and avoiding the need for injection with good compliance [16].

### Implications for further research

Our results have several implications for further research. Firstly, there was intrasubject and intersubject variability within dosing arms in the same HIF-PHIs trials, with some patients responding to lower doses and others not responding to higher doses [24]. Determinants of differences in responsiveness will be explored in future trials. Secondly, currently available HIF-PHIs targets all 3 HIF-PHDs, whereas EPO production and certain iron genes are mainly HIF-2α-controlled [51]. Thus, the development of new specific and effective PHD inhibitors are needed for the treatment of renal anemia. Thirdly, the consequences of CV events and tumor occurrence with a long period of HIF-PHIs treatment have yet to be determined. Fourthly, considering a higher dosage of ESA related risk of CV events [52,53] and HIF-PHIs only increase EPO in the physiological level, whether the target Hb level could be upregulated with HIF-PHIs treatment for anemia treatment is yet to be studied. Finally and importantly, the long term effect of HIF-PHIs on patients’ survival or CKD progression is a critical question and obviously is to be answered [54].

### Caution

Adverse events profile is an important factor in choosing therapy options. In this meta-analysis, we found that the risk of nausea and diarrhea were significantly increased after HIF-PHIs treatment on anemia in patients with CKD. Although nausea and diarrhea were generally tolerable and rarely led to drug withdrawal or trial interruption, caution is still needed before a conclusion can be made. In addition, the follow-up time of the included studies is generally short, and some side effects may not occur at the end of the experiment, such as tumor occurrence. As we know, treatment of renal anemia may be a protracted battle, even requiring lifelong medication, the influence of long term use on the transcription of other genes or off-target effects should be strictly evaluated in the future study.

### Weaknesses of this study

There were several limitations in our meta-analysis. Firstly, we only included phase II and phase III trials with a relatively small number of patients with CKD and short duration, and several studies did not report the random sequence generation, allocation concealment, and blinding of outcome assessment. Secondly, we only considered RCTs with available results, the potential differences among HIF-PHIs may reflect the design of the studies and the number of patients included. Thirdly, heterogeneity is reported in the differences in population demographics, dose, length of follow-up, kinds of HIF-PHIs, which may due to potential differences among the molecules (half-life, selectivity, etc.). Fourthly, we found no significant publication bias in Begg’s test; however, the validity of publication bias was limited. Fifthly, concomitant iron therapies in the analyzed studies are not available. The effects of these parameters on the outcome of hemoglobin are unknown. Despite these limitations, this study is the first largest meta-analysis that incorporates results from 26 RCT studies with 2804 patients using HIF-PHIs for the treatment with renal anemia.

## Conclusion

This article represents, to our knowledge, the most extensive meta-analysis appraising the effects and safety of HIF-PHIs on renal anemia. As the first-in-class small molecule drug, HIF-PHIs have been demonstrated as efficacious as rhEPO in correction of renal anemia as well as improving iron utilization, which suggested that HIF-PHIs will have great potential to serve as a new revolutionary drug in the treatment of renal anemia.

## Supplementary Material

Supplemental Material
