# Supplementary material for: Effect of hypoxia-inducible factor-prolyl hydroxylase inhibitors on anemia in patients with CKD: a meta-analysis of randomized controlled trials including 2804 patients
Source: Ren Fail. 2020 Sep 1;42(1):912–25. doi: 10.1080/0886022X.2020.1811121 (PMC7946011; doi:10.1080/0886022X.2020.1811121)

Supplementary materials

This material has been provided by the authors to give readers additional information about their work.

Supplement to: Bin W, Qing Y, Yu-Cheng H, et al. Effect of Hypoxia-inducible factor-prolyl hydroxylase inhibitors on Anemia in CKD Patients: A meta-analysis of randomised controlled trials including 2804 patients.

TABLE OF CONTENTS OF SUPPLEMENTAL MATERIAL

Search strategies for PubMed, Web of Science, Ovid Medline and the Cochrane Library database 3

PubMed: 110 results 3

Web of Science: 224 results 3

Ovid MEDLINE: 282 results 3

Cochrane Library database: 118 results 3

SUPPLEMENTARY TABLES 4

Table S1. Risk of bias of the included trials 4

Table S2. Begg's and Egger's test for publication bias testing. 4

SUPPLEMENTARY FIGURES 5

Figure S1. Forest plot for Ferritin change from baseline. 5

Figure S2. Forest plot for transferrin change from baseline. 6

Figure S3. Forest plot for TIBC change from baseline. 7

Figure S4. Forest plot for serum iron change from baseline. 8

Figure S5. Forest plot for serum TSAT change from baseline. 9

Search strategies for PubMed, Web of Science, Ovid Medline and the Cochrane Library database

We searched English language publications up to 07 October 2019 on the following databases and international and national clinical trial registries: Ovid Medline, PubMed, Web of Science and the Cochrane Library database (no date restriction), with relevant text words and medical subject headings that included all spellings of “Prolyl hydroxylase inhibitor”, “PHD inhibitor”, “hypoxia inducible factor stabilizer”, “HIF stabilizer”, “Roxadustat”, “FG-4592”, “Vadadustat”, “AKB-6548”, “Daprodustat”, “GSK127883”, “Molidustat”, “BAY 85-3934”, “Enarodustat”, “JTZ-951”, “DS-1093a” and “anemia OR anaemia”.

PubMed: 110 results

(Prolyl hydroxylase inhibitor OR PHD inhibitor OR hypoxia inducible factor stabilizer OR HIF stabilizer OR Roxadustat OR FG-4592 OR Vadadustat OR AKB-6548 OR Daprodustat OR GSK127883 OR Molidustat OR BAY 85-3934 OR JTZ-951OR DS-1093a) AND (anemia OR anaemia)

Web of Science: 224 results

(Prolyl hydroxylase inhibitor OR PHD inhibitor OR hypoxia inducible factor stabilizer OR HIF stabilizer OR Roxadustat OR FG-4592 OR Vadadustat OR AKB-6548 OR Daprodustat OR GSK127883 OR Molidustat OR BAY 85-3934 OR JTZ-951OR DS-1093a) AND (anemia OR anaemia)

Ovid MEDLINE: 282 results

(Prolyl hydroxylase inhibitor) OR (PHD inhibitor) OR (hypoxia inducible factor stabilizer) OR (HIF stabilizer) OR Roxadustat OR FG-4592 OR Vadadustat OR AKB-6548 OR Daprodustat OR GSK127883 OR Molidustat OR (BAY 85-3934) OR JTZ-951OR DS-1093a

Cochrane Library database: 118 results

("Prolyl hydroxylase inhibitor" OR "PHD inhibitor" OR "hypoxia inducible factor stabilizer" OR "HIF stabilizer" OR "Roxadustat" OR "FG-4592" OR "Vadadustat" OR "AKB-6548" OR "Daprodustat" OR "GSK127883" OR "Molidustat" OR "BAY 85-3934" OR "JTZ-951" OR "DS-1093a") AND ("anemia" OR "anaemia")

SUPPLEMENTARY TABLES

Table S1. Risk of bias of the included trials

Study,year	Randomization	Allocation concealment	Blinding of participants and staff	Blinding of outcome assessors	Incomplete outcome data	Selective outcome reporting	Other sources of bias
Tadao Akizawa 2017	Low	Low	Low	Low	Low	Low	Low
Louis Holdstock 2019	Low	Low	High	Low	Low	Low	Low
Amy M. Meadowcroft 2018	Low	Low	High	Low	Low	Low	Low
Louis Holdstock 2016	Low	Low	High	Low	Low	Low	Low
Edouard R. Martin 2017	Low	Low	Low	Low	Low	Low	Low
Pablo E. Pergola 2016	Low	High	High	Low	High	High	Low
Tadao Akizawa 2019	Low	Low	Low	Low	Low	Low	Low
Anatole Besarab 2015	Low	Low	High	Low	Low	Low	Low
Nan Chen 2017	Low	Low	Low	Low	Low	Low	Low
Chen 2019	Low	High	High	Low	Low	Low	Low
Chen 2019	Low	Low	High	Low	Low	Low	Low
Provenzano 2016	Low	High	High	Low	Low	Low	Low
Akizawa 2019	Low	High	High	Low	Low	Low	Low
Akizawa 2019	Low	Low	Low	Low	Low	Low	Low
Deven V. Parmar 2019	Low	Low	Low	Low	Unclear	Low	Low
Iain C. Macdougall, 2018	Low	High	High	Low	Low	Low	Low
Tadao Akizawa 2019	Low	High	High	Low	Low	Low	Low

Evaluate using the Cochrane Bias Risk Assessment Tool. The method quality of the included trials was generally moderate to good.

Table S2. Begg's and Egger's test for publication bias testing.

Parameters	P for Begg's test	P for Egger's test
Hemoglobin	0.530	0.636
Hepcidin	0.369	0.812
Ferritin	0.778	0.375
Transferrin	0.761	0.770
Total iron binding capacity	0.023	0.004
Iron	0.965	0.788
Transferrin saturation	0.788	0.643

The Begg rank correlation test and Egger rank correlation test indicated no evidence of publication bias except for total iron binding capacity.

SUPPLEMENTARY FIGURE LEGENDS

Figure S1. Forest plot for Ferritin change from baseline. Positive value in mean difference of ferritin change represent a significant lower level of ferritin in PHI group than in the control group at the end of the treatment of PHI. Abbreviations and definitions: MD, mean difference; CI, confidence interval; HIF-PHI, hypoxia inducible factor-Prolyl hydroxylase inhibitor.

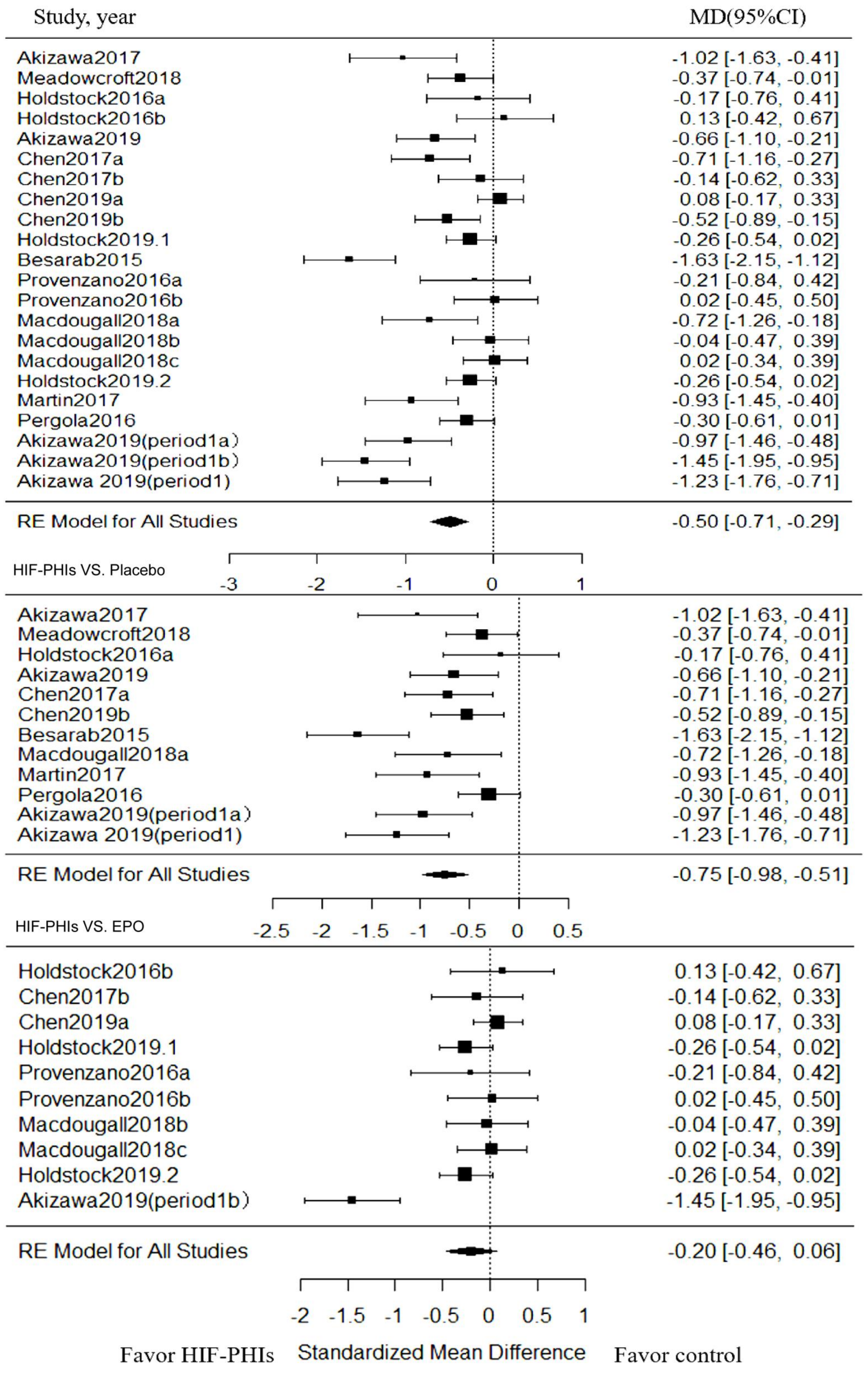

Figure S2. Forest plot for transferrin change from baseline. Positive value in mean difference of transferrin change represent a significant lower level of transferrin in PHI group than in the control group at the end of the treatment of PHI. Abbreviations and definitions: MD, mean difference; CI, confidence interval; HIF-PHI, hypoxia inducible factor-Prolyl hydroxylase inhibitor.

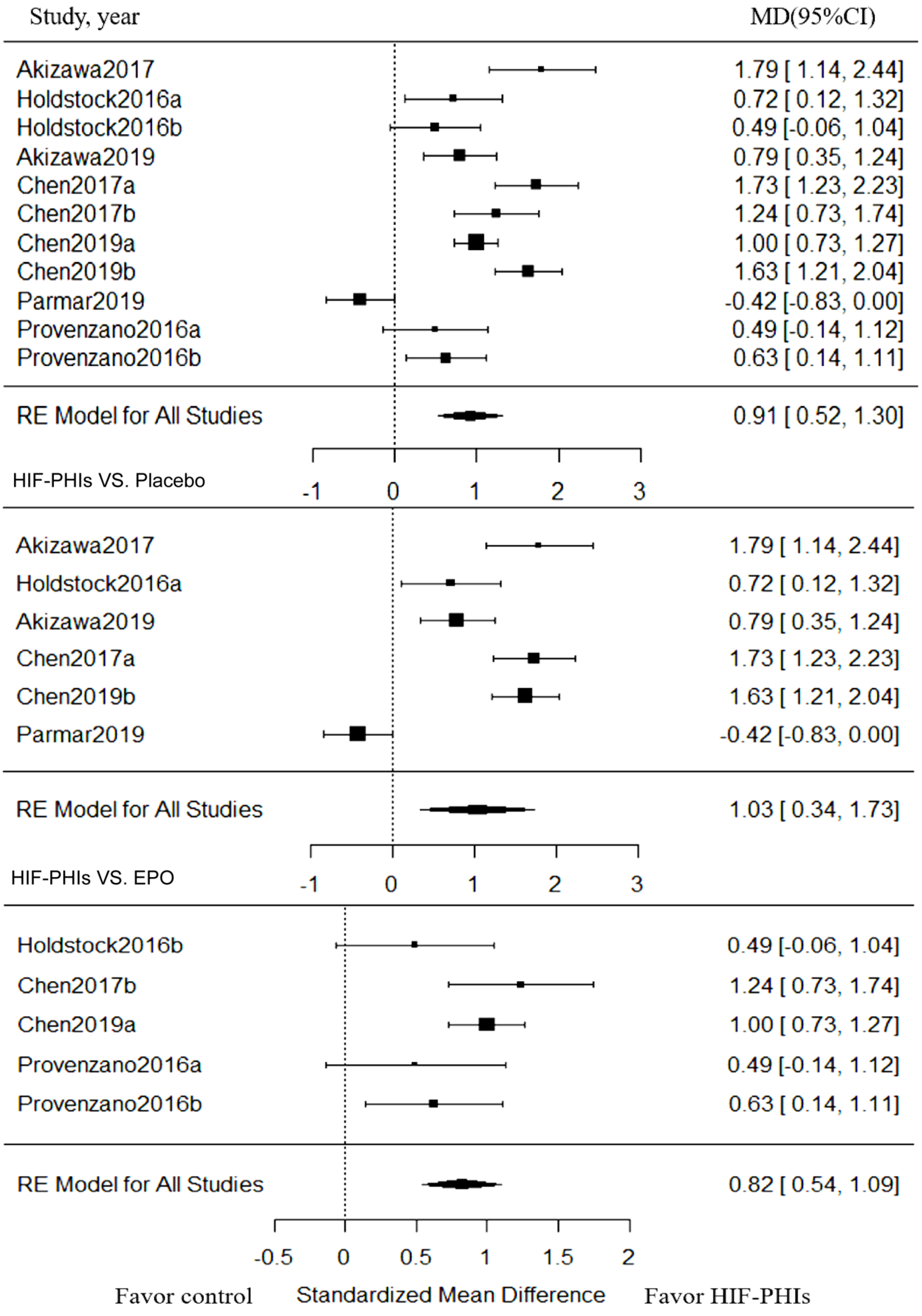

Figure S3. Forest plot for TIBC change from baseline. Positive value in mean difference of TIBC change represent a significant higher level of TIBC in PHI group than in the control group at the end of the treatment of PHI. Abbreviations and definitions: MD, mean difference; CI, confidence interval; HIF-PHI, hypoxia inducible factor-Prolyl hydroxylase inhibitor; TIBC, Total iron binding capacity.

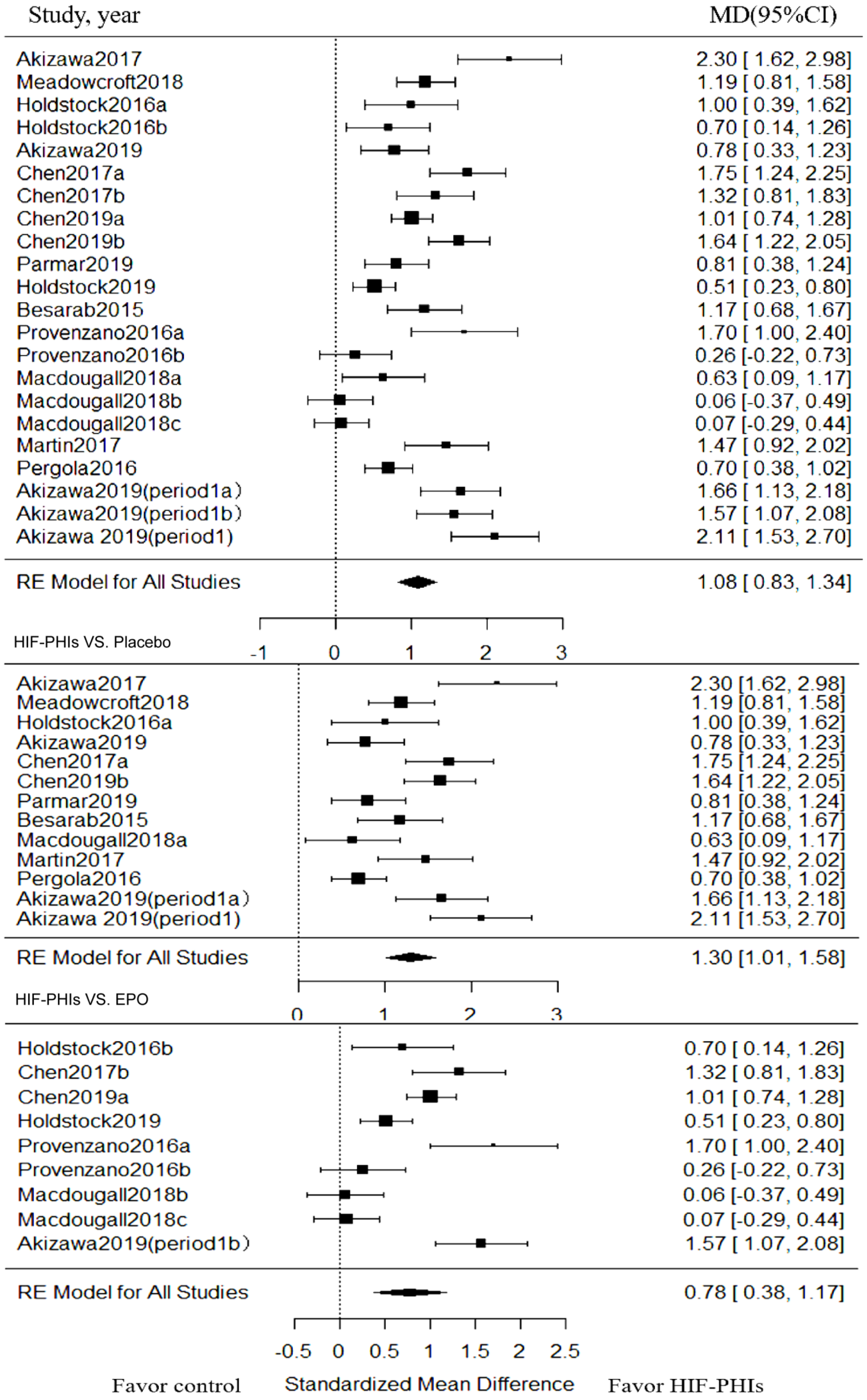

Figure S4. Forest plot for serum iron change from baseline. Positive value in mean difference of iron change represent a higher level of iron in PHI group than in the control group at the end of the treatment of PHI. Abbreviations and definitions: MD, mean difference; CI, confidence interval; HIF-PHI, hypoxia inducible factor-Prolyl hydroxylase inhibitor.

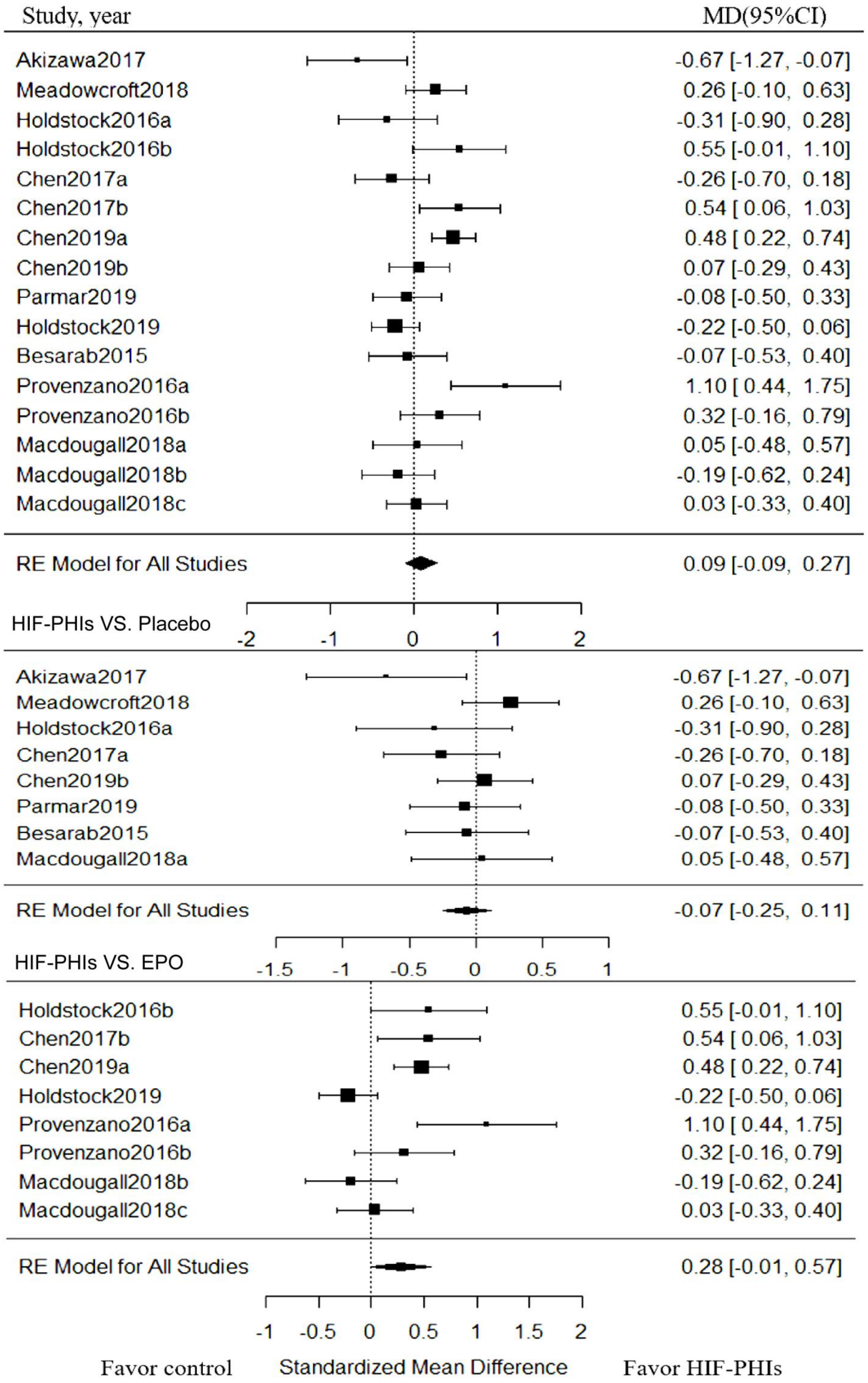

Figure S5. Forest plot for serum TSAT change from baseline. Positive value in mean difference of TSAT change represent a lower level of iron in PHI group than in the control group at the end of the treatment of PHI. Abbreviations and definitions: MD, mean difference; CI, confidence interval; HIF-PHI, hypoxia inducible factor-Prolyl hydroxylase inhibitor; TSAT, Transferrin saturation.

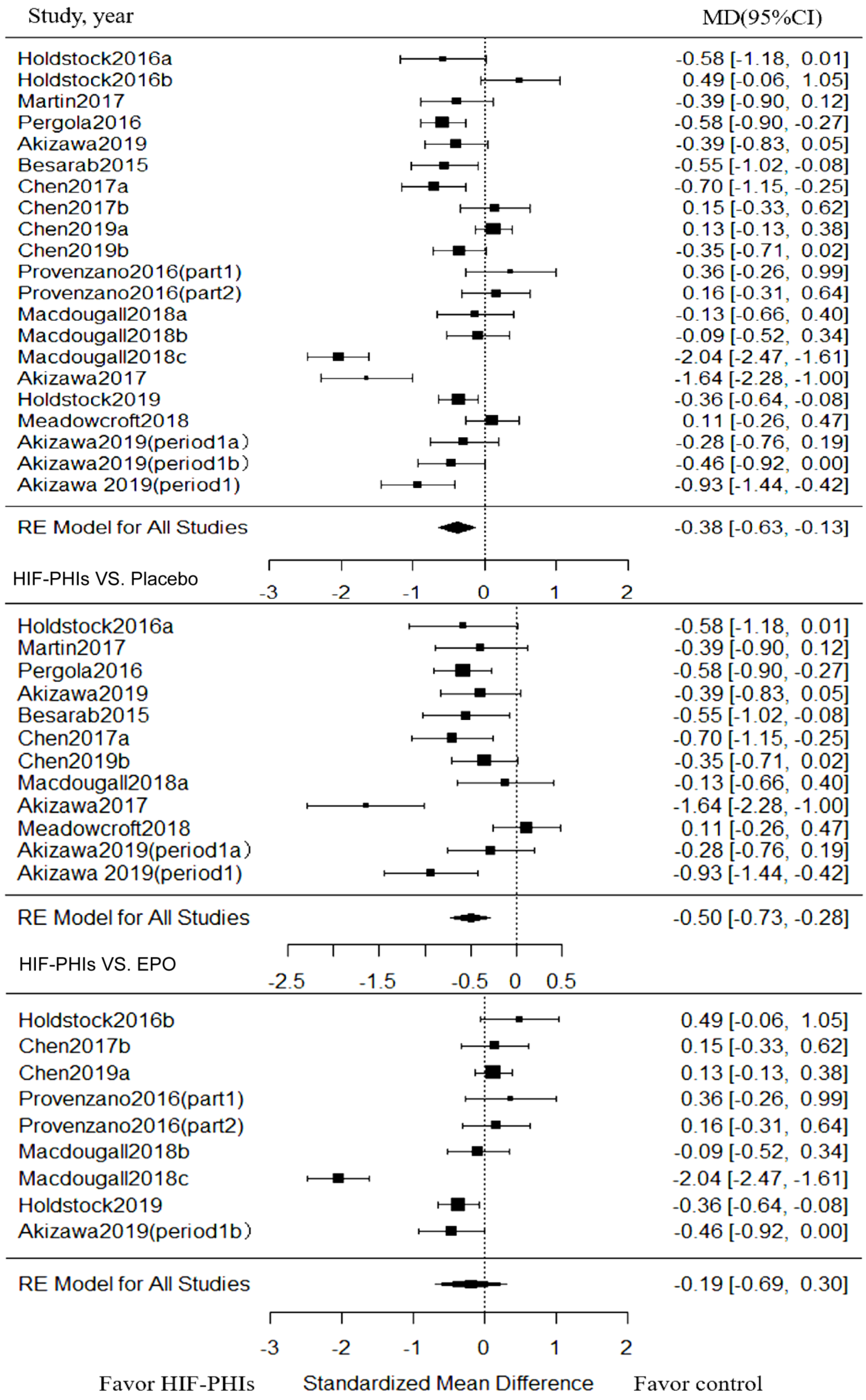

Supplement: Supplemental Material [file IRNF_A_1811121_SM0237.pdf]
